# Syphilis of the Testis

**Published:** 1917-07

**Authors:** A. Ravogli

**Affiliations:** Cincinnati, Ohio


### SYPHILIS OF THE TESTIS.

By A. Ravogli, M.D., Cincinnati, Ohio

Syphilis affects the organs of the elaboration of the semen not so frequently, as it would appear from a speculative induction. That the semen of the sphilitic contains the spirochaeta, it would seem from the invasion of the ovum at the time of the fecundation. The spirochaeta, however, does not injure the stroma and the organ very often. Amongst my patients of private practice I cannot see that syphilitic orchi-epididymitis occurs more than 3 per cent., but, in hospital practice probably more than 5 per cent.

Hospital practice offers orch-epididymitis of a mixed kind. In those cases with syphilis, a gonorrheic attack determines the gonorrheal epididymitis, which after awhile is the cause of the syphilitic affection of the testicle.

In my experience I must say that if a syphilitic is infected with gonorrhea he may get a gonorrheal orchi-epididymitis purely gonorrheic. The injury, however, which is done to the testis and to the epididymis by the gonorrheal inflammatory process, is capable of arousing the syphilitic process, and when the gonorrhea is better the testicle remains swollen, hard, globular, irregular, with some serous affusion into the vaginalis, which lasts for a long time, with permanent injury of the generative organs and generative function. In a word, it is a case of pars minoris resistentiae. The gonorrheal process affects the epididymis and, at time, the testis in an acute way; in a short time the process is diminished, but a chronic infiltration remains. This condition is often the beginning of the syphilitic orchi-epididymitis, which follows the somatic injuries produced by the acute gonorrheal infection.

My views will probably be somewhat different from others, who see a line of demarcation between a gonorrheal orchi-epididymitis and a syphilitic orchi-epididymitis, merely in the assumption that a man who has syphilis could not be affected with gonorrhea and then with gonorrheal orchi-epididymitis.

Syphilitic affection may be limited to the epididymis, to the testicle, to the vaginalis, or may affect the whole apparatus, in the form of a simple inflammatory form in early syphilis. In late syphilis the whole testicle may be invaded in a gummous form.

Epididymitis in the secondary stage of syphilis was first described by Drou (1), which was after a while confirmed by Fournier, Jullien and others. The affection makes its appearance at the late secondary stage in the form of an indurated point at the head of the epididymis of the size of a hazel-nut. In some cases it is indolent, though it is usually accompanied by acute pain and tenderness. The inflammation spreads to the neighboring structures of the testicles and cord, which are involved together with the epididymis. The tunica vaginalis is also involved, and gradually is filled with an abundant hydrocele. In many cases extensive induration of the epididymis and of the testis is accompanied with considerable hydrocele.

Since the first observations of Oelnitz (2) and Ozenne (3) on syphilitic pachivaginalitis with effusion of bloody serum, it has been recognized that this disease is a hemorrhagic pachivaginalitis of syphilitic origin.

In the secondary period affections of the testis are rare, but more frequently occur in the late stage of the disease.

It may occur in a duffuse form as an interstitial orchitis, a chronic inflammation of the connective elements, of the septa of the organ, which Ricord called albuginitis. It is a specific inflammatory process of the interstitial connective tissues, chronic in its course, which causes pressure on the delicate canaliculi of the secretory organ, with consequent injury of the glandular parenchyma and its function, causing sometimes atrophy of the testis. The delicacy of the glandular portion with its system of tubuli, is in contrast with the dense fibro-elastic tissue that composes the frame-work of the testis capsule, mediastinum and interlobular septa. The connective tissue which is between the spaces of the seminiferous tubuli is loose, and consists of delicate white connective tissue with a few elastic fibres. Blood and lymph vessels and nerves are abundant in the vicinity of the mediastinum. The spermatic and the deferential arteries with their anastomoses in form of twigs reaching the mediastinum penetrate the albuginea and the septa surrounding the seminiferous tubules. From the anatominal structure it is easy to understand that the typical, initial, histological alterations are to be found in the walls or around the blood vessels, as a proof that the spirochaeta finds its way through the blood circulation. The process causes the connective tissues to proliferate and infiltrate the tubuli of the secreting organs which consequently are impaired in their function. When the inflammatory process has completed its course, when the infiltrated elements are reabsorbed the fibrous tissues assume a sclerotic hardness, shrink, and compressing the blood vessels, cut off the supply of nutrition to the glandular elements, and the testicle together with the epididymis is converted into a hard atrophic nodule, destitute of any function.

This form of syphilitic inflammatory process is an orchitis of an intertstitial type. The induration comes from the connective tissues forming the septa, running along the tubuli, and the small seminal ducts. It causes at first an enlargement of the gland, then contraction and atrophy. In most of my cases the surface of the testicle was smooth, showing a process equally diffused. Some cases, when the process is confined to circumscribed parts, may give the impression of hard nodules. In these cases small exudations into the tunica vaginalis which is also thickened, may take place. Keyes referred to six cases, and Fuller to two cases where an extensive induration of the epididymis and of the testis was accompanied by considerable hydrocele.

In my private practice I had occasion to treat a young man who had suffered a sever case of syphilis for over two years. He began to complain of a swollen testicle, which gradually reached an enormous size. The surface of the testis was uneven, the epididymis could scarcely be distinguished from the testis. It was a uniform mass, elongated in the shape of a sausage extending to the spermatic cord. On account of the undulating sensation produced by the serous effusion, it was tapped, but only a small quantity of brownish, bloody serum was obtained.

Syphilitic orchitis, especially when coming a few months after the infection, may be bilateral. It is accompanied by little pain and the swelling is diffused over the testicles together with the epididymis. Usually syphilitic orchitis is a late manifestation of syphilis, the enlargement of the testicle takes place slowly, and rather than a sense of pain, the patient complains of a sense of weight in the scrotum.

Fortunately this form of orchi-epididymitis responds promptly to the antisyphilitic treatment. Unguent hydrargyri locally, mercurial intramuscular injections and potassium iodide by the mouth, has in many cases returned the testicle to the normal size without permanent injury.

Gumma of the testicle, the orchitic gummosa of Virchow, rarely occurs, and also rarely suppurates. It is usually reabsorbed, causing sclerosis and contraction of the affected tissues. In consequence, the segregating part of the gland rerely escapes destruction. This causes diminution of the spermatic secretion or even total aspermia, with lack of desire for sexual relation or even total impotence. After some months the whole mass is reduced to a shapeless rudiment of the testicle.

In some cases the gummatous infiltration of the testicle forms a tumor involving all the coverings of the testicle together with the skin, which softens and perforates. The gumma so ulcerated, by proliferation of the connective tissues, sticks out as a granulating mass. This condition has been named fungus testiculi syphiliticus. In the beginning it is accompanied with dull pain, which gradually subsides. It takes the appearance of a fleshy tumor, protruding from the skin, narrowed at its base, red, cyanotic, exuding pus, and covered with necrotic detritus.

The fungus of the testis left to itself lasts for some time, but gradually becomes smaller, partly by absorption, mostly by sloughing of the necrotic parts.

The general condition of the patient is not involved with the infection of the testicle on account of its indolent course, but it usually produces great depression of the mind, when it is known that the organs of the generation have been attacked. I have seen patients affected with syphilitic orchitis who complained of obstinate insomnia, caused by the gloomy thoughts of remaining impotent.

In the case of suppurated gumma of the testis, the prolonged suppuration and the resulting fever weakens the patient. The cicatrization follows according to the treatment employed.

In my hospital practice I had a case of syphilitic gumma of the testis with abundant suppuration. The clinical appearance could have been mistaken for tuberculosis. The man was very much run down from the long standing suppuration. The principal simus was incised, opening from the upper segment of the scrotum to the bottom. With a curette all the suppurating and necrotic masses were removed, leaving the testicle and the epididymis which it was hoped to save. The mass of detritus was examined for tubercle bacilli with negative results. The Wassermann was slightly positive. The wound of the scrotum and testicle was left open, and packed with iodoform gauze. As at that time salvarsan could not be obtained, internally Zittmann decoction and potassium iodide were given. In not a long time the patient made a quick recovery and left the hospital in excellent condition.

Another recent case of syphilis of the testis was in a colored man, 26 years old, who strong and well built physically, denied any syphilitic infection. He had been in the medical ward in March, 1916, for bronchitis, and in June, 1916, had had pleurisy. In 1912, he had been affected with gonorrhea and epididymitis. Two months ago his right testicle began to swell to form an even mass. Epididymis and testicle could not be distinguished; the vaginalis was filled with serum. Wassermann test was positive. Diagnosis syphilitic orchi-epididymitis. The vaginalis was opened, the fluid removed and drained. The patient was treated with grey oil injections and potassium iodide internally. Two weeks later the wound was healed up, the patient was greatly improved and asked to be released from the service.

It would be superfluous to cite more cases and more histories of patients, which greatly resemble each other. In many cases the patient denies infection, but the Wassermann test reveals the true nature of the affection. In some cases, however, the Wassermann may be negative on account, very likely of a weakened condition of the patient, as I have had in more than one case recently under my care, but with positive signs of progressed syphilis. The points to make clear are the differentiation between gonorrheal epididymitis, tubercular and syphilitic orchi-epididymitis.

Gonorrheal epididymitis is the most common form of all diseases of the testicle. It is usually acute in character, accompanied sometimes by fever, and with an unbearable pain. It is the result of the gonnorheal attack of the posterior urethra. It ends by involution, rarely by abscess. In some cases it may remain subacute or assume a chronic course. It is mostly unilateral; at times it may affect both testicles in an alternating way, very rarely simultaneously.

It is easy to differentiate a syphilitic affection of the testicle from a gonorrheal epididymitis. The absence of pain in a syphilitic testicle clearly contrasts with the extreme pain and tenderness of the gonorrheal epididymitis. In the last the spermatic cord is also involved and the scrotum of the affected part is red, tender and swollen. There will be found discharge of a gonorrheal nature from the urethra, or the patient will admit that it was present for a time, or better still the urine will be turbid, showing muco-pus and shreds. Some difficulty can be found when a gonorrheal epididymitis in a syphilitic assumes a chronic course, and very likely starts a syphilitic orchi-epididymitis. In the genuine syphilitic orchitis the affection begins without pain, the swelling of the testicle is the principal symptom, which only causes a sense of weight. In syphilitic orchitis the epididymis can scarcely be distinguished. It forms an uneven mass, made up of nodules formed in the testicle.

Some difficulty may arise in a differential diagnosis between tuberculosis and syphilis. In most of the cases, tuberculosis affects the epididymis. It affects this organ in the epithelium and in the walls of the canals as grey-whitish foci of infiltration, independent of each other, ranging from the size of a millet seed to that of a split pea, or even to that of a hazelnut. These nodules are at first firm and hard to the touch, but in a few weeks are softened. The skin is bluish red in color, and shows small prominences which break down and give issue to pus. There is left a fistula which, through a tortuous path, leads to the place of infiltration. From the fistula thin pus exudes, which in many cases shows presence of the tubercle bacilli. It heals up now and then, but in a short time opens again discharging the accumulated pus. Tuberculosis of the epididymis and testis causes a symptomatic hydrocele, and later on the serous membrane is also affected with tubercles. The process is usually accompanied with fever, especially in the evening. The patient gets weak on account of the continuous suppuration. In many instances tuberculosis of the testicle is accompanied by pulmonary tuberculosis.

There will be no difficulty in differentiating a syphilitic orchitis from a tubercular testis, taking under consideration the seat of the affection, the purulent disintegration of the infiltrated foci, and the presence of tubercle bacilli.

In this short paper I will not mention other forms of orchitis, which can be traumatic, or of urethral origin, or metastatic, as we find in mumps, epidemics of variola, typhus, acute rheumatism, pneumonia, malaria. All these kinds of orchitis are of an acute type, end in suppuration and show the presence of the bacilli, as the Eberth bacillus in typhus.

I have dealt somewhat longer on the differential diagnosis between tuberculosis and syphilis of the testis for the only reason that I have seen several patients who have had a testicle removed for tuberculosis when it was syphilitic, and the mutilation could have been prevented by a definite diagnosis.

The prognosis of syphilis of the testis has no importance for the life of the patient, only for the life of the organ. In many cases the destruction of the testicle will follow and if bilateral in all probabilities the individual will remain impotent.

Treatment: The general treatment of syphilis embraces salvarsan, alternated with mercury, until cure, thus preventing any late manifestations. Treatment has to be continued until the Wassermann test is negative and no symptoms can be seen.

When the testis is affected with syphilis, locally unguent, cinereum is applied, and for general treatment mercurial injections with grey oil, or salicylate of mercury or better still calomel. Internally potassium iodid to the highest possible tolerance. Between the mercurial injections one or two injections of salversan 0.6 will hasten recovery.

When broken down gummata have caused abscesses, with sinuses or hydrocele, the sinuses have to be widely opened and the serum or pus drained. The broken down gumma has to be scraped with the curette, the necrotic tissues removed and all has to be packed with iodoform gauze. I can cheerfully state that in the most of my cases I have obtained satisfactory results. In reference to the generative functions I have had a chance to follow onl ytwo cases, one had syphilitic testicle after he had married, and after the treatment had a child, and another after being treated got married and has children—The Urologic and Cutaneous Review.

